# A Rare Case of Low-Grade Endometrial Stromal Sarcoma: Diagnostic Insights and Therapeutic Considerations

**DOI:** 10.7759/cureus.88983

**Published:** 2025-07-29

**Authors:** Soufia El Ouardani, Hind Chibani, Hanane Hadj Kacem, Sami Aziz Brahmi, Said Afqir

**Affiliations:** 1 Medical Oncology, Mohammed VI University Hospital, Oujda, MAR; 2 Medical Oncology, Faculty of Medicine and Pharmacy of Oujda, Mohamed First University, Oujda, MAR; 3 Radiology, Mohammed VI University Hospital, Oujda, MAR; 4 Radiology, Faculty of Medicine and Pharmacy of Oujda, Mohamed First University, Oujda, MAR

**Keywords:** good prognosis, immunohistochemistry staining, low-grade endometrial stromal sarcoma, pathology, surgery

## Abstract

Low-grade endometrial stromal sarcoma (LGESS) is a rare malignant tumor of the uterus, often misdiagnosed due to its indolent progression and nonspecific clinical presentation. We report the case of a 49-year-old perimenopausal woman who presented with abnormal uterine bleeding and pelvic pain. Pelvic ultrasound and MRI revealed a heterogeneous uterine mass. The patient underwent a total hysterectomy, and definitive histopathology confirmed the diagnosis of LGESS. This case underscores the importance of including LGESS in the differential diagnosis of abnormal uterine bleeding in perimenopausal women. Early diagnosis and complete surgical excision remain the cornerstone of treatment, and long-term follow-up is crucial due to the risk of late recurrence.

## Introduction

Low-grade endometrial stromal sarcoma (LGESS) is a rare mesenchymal tumor, accounting for 6-20% of all uterine sarcomas [1]. It primarily affects premenopausal women [2]. Diagnosis relies on histological evaluation, which must be interpreted with caution to distinguish it from other entities such as leiomyoma [3]. The standard treatment is surgical, with total hysterectomy being the mainstay of care [4]. The role of additional treatments - including lymph node dissection, radiotherapy, and medical therapies such as chemotherapy and hormone therapy - remains a subject of debate [4]. Compared to other types of endometrial sarcoma, low-grade stromal sarcoma is associated with a more favorable prognosis, with an overall five-year survival rate exceeding 90% [2].

## Case presentation

**Figure 1:**
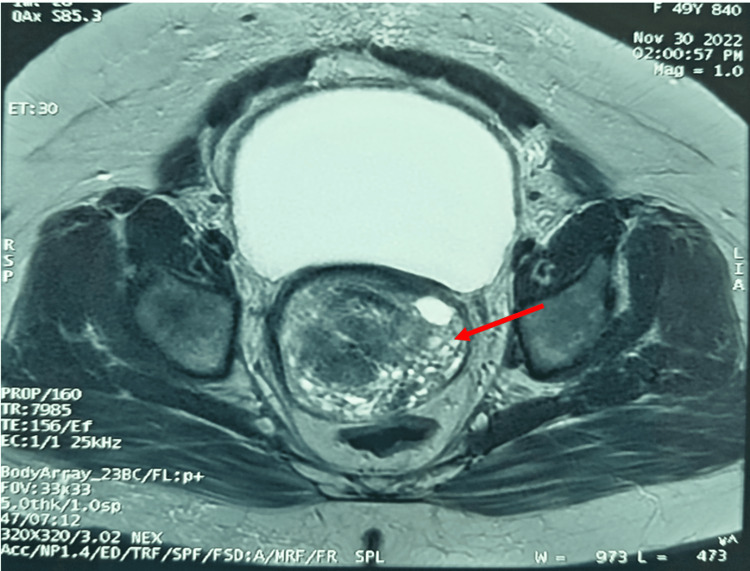
Axial T2-weighted pelvic MRI showing a large tumor mass occupying the uterine lumen (red arrow), consistent with a genital tract neoplasm

A 49-year-old perimenopausal patient with a history of diabetes managed with oral antidiabetic medication was admitted to the oncology hospital for evaluation of menometrorrhagia. On admission, clinical examination revealed a hemodynamically stable patient with good performance status (PS = 1) and no clinical signs of anemia. Vaginal examination identified a cervical mass protruding into the vaginal canal. Pelvic ultrasound revealed an endometrial thickening measuring 32 mm, along with a cystic image extending from the umbilicus, suggestive of a distended bladder. To further characterize the ultrasound findings, a pelvic MRI was performed, which demonstrated a large tumor process involving the genital tract and occupying the uterine, cervical, and vaginal lumens (Figure 1).

**Figure 2:**
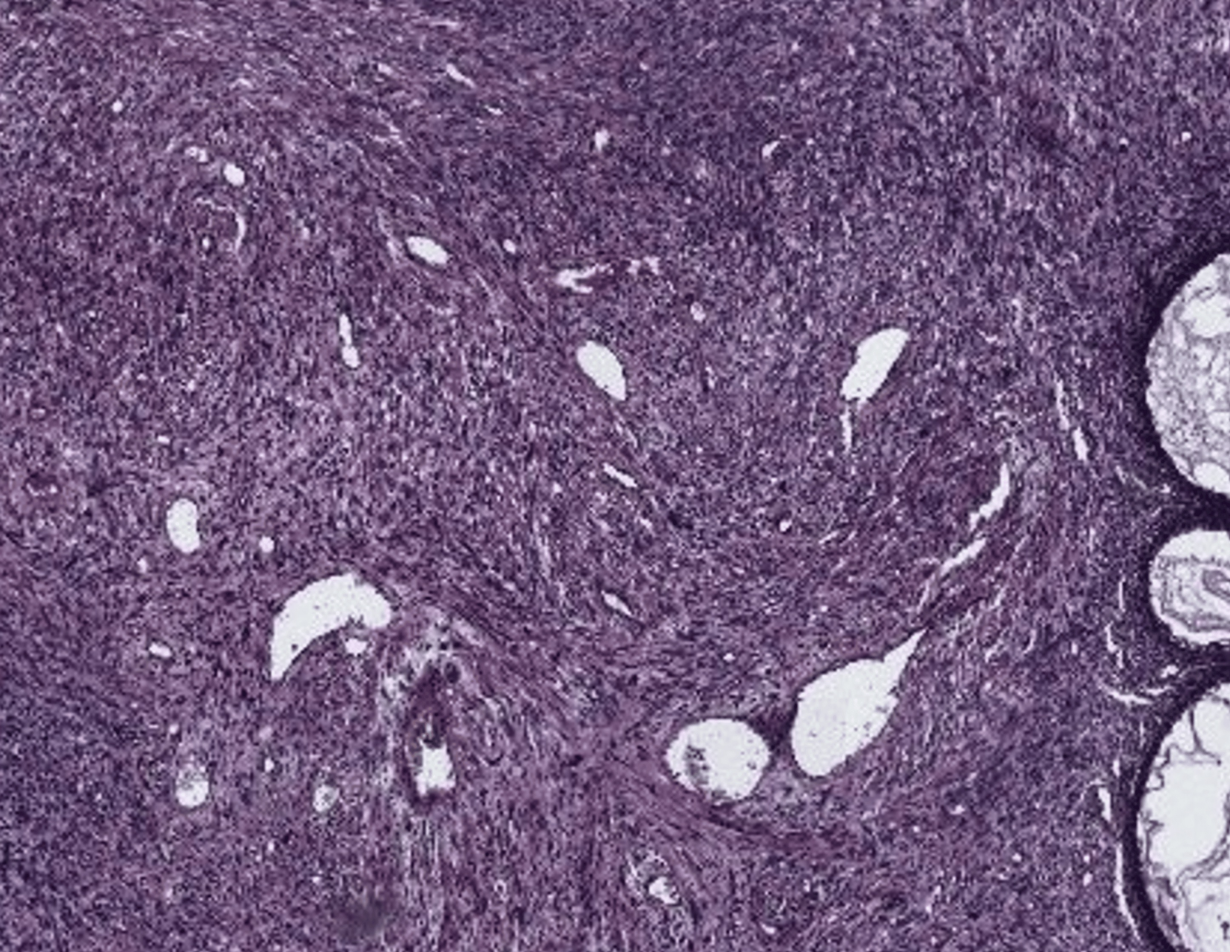
Photomicrograph of the tumor (H&E, ×10) The image shows a cellular proliferation arranged in sheets, predominantly composed of spindle-shaped cells. The nuclei appear enlarged with irregular contours, dense chromatin, and thickened nuclear membranes. Atypical mitotic figures are infrequent.

**Figure 3:**
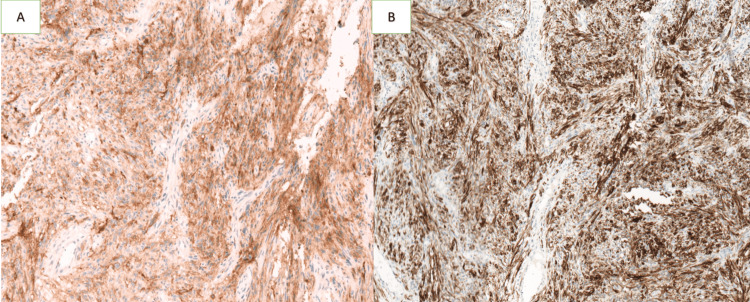
Immunohistochemical staining showing positive expression of tumor cells with anti-CD10 (A) and anti-Desmin (B) antibodies (magnification ×10)

**Figure 4:**
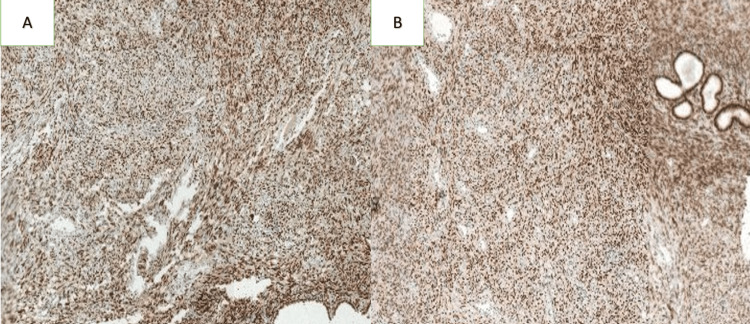
Immunohistochemical staining demonstrating positive expression with anti-estrogen receptor (A) and anti-progesterone receptor (B) antibodies (magnification ×10)

The initial biopsy revealed a spindle-cell proliferation. A thoracic and abdominopelvic CT scan was performed, which showed no evidence of distant metastases. The patient underwent an enlarged colpohysterectomy, and histopathological examination, supplemented by immunohistochemistry (IHC), confirmed a diagnosis of LGESS measuring 9 cm in length. Surgical margins were negative, and there was no infiltration of the paracervical parameters (Figure 2). IHC demonstrated positive staining for anti-CD10, anti-desmin, anti-vimentin, anti-estrogen, and anti-progesterone antibodies (Figure 3, Figure 4).

After a discussion at a multidisciplinary consultation meeting, the patient was placed under surveillance. She has remained well-controlled, with a follow-up period of 45 months to date.

## Discussion

The World Health Organization classifies endometrial stromal tumors into four histological types: endometrial stromal nodules, low-grade stromal sarcomas, high-grade stromal sarcomas, and undifferentiated sarcomas [5]. LGESS is a rare subtype, accounting for less than 1% of all endometrial cancers [1]. It most commonly affects premenopausal and perimenopausal women [2]. The uterine body and cervix are the primary sites of involvement, while the ovary is the most frequently reported extrauterine location [6]. LGESS typically follows an indolent clinical course, and distant metastases are rare [6].

Clinical symptoms, such as menometrorrhagia and pelvic pain, are nonspecific and overlap with those of other gynecological conditions, often leading to delayed diagnosis. LGESS presents a diagnostic challenge and is frequently misinterpreted as a benign lesion, such as uterine leiomyoma [3]. The differential diagnosis includes leiomyoma, adenomyosis, endometrial carcinoma, and other sarcomas [7].

Pelvic ultrasound is usually the first-line imaging modality and may reveal an irregular mass infiltrating the endometrium or myometrium, sometimes with necrotic, hemorrhagic, or cystic areas [8]. Pelvic MRI is particularly useful for evaluating uterine and ovarian lesions [9]. On MRI, LGESS may appear as a well-defined myometrial mass, an irregular lesion extending from the endometrium into the myometrium, or, less commonly, as a polypoid mass within the uterine cavity [10].

Definitive diagnosis is based on histopathological examination, which typically shows a proliferation of small, round neoplastic cells resembling proliferative-phase endometrial stromal cells [11]. Cytonuclear atypia is rare, and the proliferation index is generally low [11]. IHC usually demonstrates positivity for CD10, along with possible expression of hormone receptors, desmin, vimentin, and muscle-specific actin [12]. Molecular analysis frequently reveals the juxtaposed with another zinc finger gene 1 - suppressor of zeste 12 (JAZF1-SUZ12) fusion gene, which is the most common genetic alteration in LGESS [12,13].

The cornerstone of treatment is surgical resection, typically involving total hysterectomy with bilateral adnexectomy for stage I disease. Ovarian preservation may be considered in premenopausal women [2]. The role of adjuvant therapy, such as hormone therapy and radiotherapy, remains controversial and should be evaluated through multidisciplinary consultation, particularly for stage II-IVA disease and beyond. The benefit of these treatments in terms of survival has yet to be clearly established [14].

## Conclusions

LGESS is a rare uterine malignancy with a slow-growing clinical course and the potential for late recurrence. Accurate diagnosis relies on a combination of morphological features, immunohistochemical profiling, and molecular identification of the JAZF1-SUZ12 fusion gene. Complete surgical resection remains the primary treatment, while long-term follow-up is essential due to the risk of delayed relapse. Further research into the molecular pathways involved in LGESS may help identify novel therapeutic targets and improve patient outcomes.
